# Research progress of the engagement of inorganic nanomaterials in cancer immunotherapy

**DOI:** 10.1080/10717544.2022.2086940

**Published:** 2022-06-24

**Authors:** Tingwei Peng, Tianzhao Xu, Xinghui Liu

**Affiliations:** a Postgraduate Training Base in Shanghai Gongli Hospital, Ningxia Medical University, Pudong New Area, China; b Shanghai Qiansu Biological Technology Co., Ltd, Pudong New Area, China; c Department of Clinical Laboratory, Gongli Hospital, School of Medicine, Shanghai University, Shanghai, China

**Keywords:** Inorganic nanomaterials, tumor immunity, cancer vaccine, tumor microenvironment, chemoimmunotherapy

## Abstract

Cancer has attracted widespread attention from scientists for its high morbidity and mortality, posing great threats to people’s health. Cancer immunotherapy with high specificity, low toxicity as well as triggering systemic anti-tumor response has gradually become common in clinical cancer treatment. However, due to the insufficient immunogenicity of tumor antigens peptides, weak ability to precisely target tumor sites, and the formation of tumor immunosuppressive microenvironment, the efficacy of immunotherapy is often limited. In recent years, the emergence of inorganic nanomaterials makes it possible for overcoming the limitations mentioned above. With self-adjuvant properties, high targeting ability, and good biocompatibility, the inorganic nanomaterials have been integrated with cancer immunotherapy and significantly improved the therapeutic effects.

Up to now, cancer has posed a serious threat to human survival due to its increasing incidence and mortality rate, which makes the emergence of comprehensive, systematic, and effective treatment modalities imminent (Siegel et al., 2019). In the early stage of cancer, the treatment strategy was mainly based on traditional treatment modes such as surgery, radiotherapy, chemotherapy, etc. However, as the research progressed, it was found that such treatment modes would lead to tumor drug resistance, systemic toxic side effects secondary to the off-target phenomenon of chemotherapy drugs, tumor recurrence, and metastasis due to postoperative tumor residual foci, etc. Hence, a series of new treatment modes represented by gene therapy and immunotherapy have been developed, which can significantly compensate for the shortcomings of traditional treatment modes (Wang et al., 2021).

Facing the invasion of tumor cells, the body has the self-protection mechanism of anti-tumor immune response and can activate the anti-tumor immune response through the immune surveillance function of the immune system in time to clear the tumor cells in the body. In this process, immune cells such as antigen-presenting cells (APC), T lymphocytes, and cytokines such as IL-4, IL-1β, and TNF-α play very important roles. First, the antigen-presenting cells (especially the specialized antigen-presenting cells DC cells) recognize the specific antigen presented by the tumor cells, and then internalize the antigen into the cytoplasm through a series of internalization mechanisms, then enter the intracellular body or lysosome through TLR-like receptor-mediated pathway for processing into short peptides, etc., and then bind to the major histocompatibility complex I (MHC-I) or II (MHC-II) to deliver the antigen information to the primary T cells. Subsequently, under the synergistic effect of co-stimulatory molecules, presented antigen information, and cytokines secretion, the primary T cells are activated and exert their anti-tumor effects (Heath & Carbone, 2001; Gong et al., 2021). However, the formation of tumor immunosuppressive microenvironment limits the further clinical application of immunotherapy, which is manifested at the cellular level by tumor infiltration of Treg (regulatory T cells), TAM (tumor-associated macrophages), and MDSC (myeloid-derived suppressor cells), and at the metabolic level by hypoxia, adenosine accumulation and immunosuppressive cytokine secretion, accompanied by upregulation of PD-1 or CTLA-4 expression on the T cell surface and PD-L1 expression on cell membranes of DCs or cancer cells (Rabinovich et al., 2007). In addition, the short half-life of tumor antigens and the loss of antigen expression due to gene mutations further weaken their immunogenicity and limit the efficacy of tumor immunotherapy (Wang et al., 2021; Gong et al., 2021).

In recent years, the emergence and development of nanomaterials have gained some momentum in improving the therapeutic effects of tumor immunotherapy. Inorganic nanomaterials are used as nanoscale carriers to maximize tumor immunotherapy by carrying a series of tumor antigens and immunomodulators and targeting specific tumor sites for stable and controlled drug release (Gong et al., 2021). At the same time, they have high biocompatibility and low cellular and systemic toxicity (Hashemi et al., 2020). Therefore, the use of inorganic nanomaterials for tumor immunotherapy has broad clinical application prospects (Figure 1).

**Figure 1.**
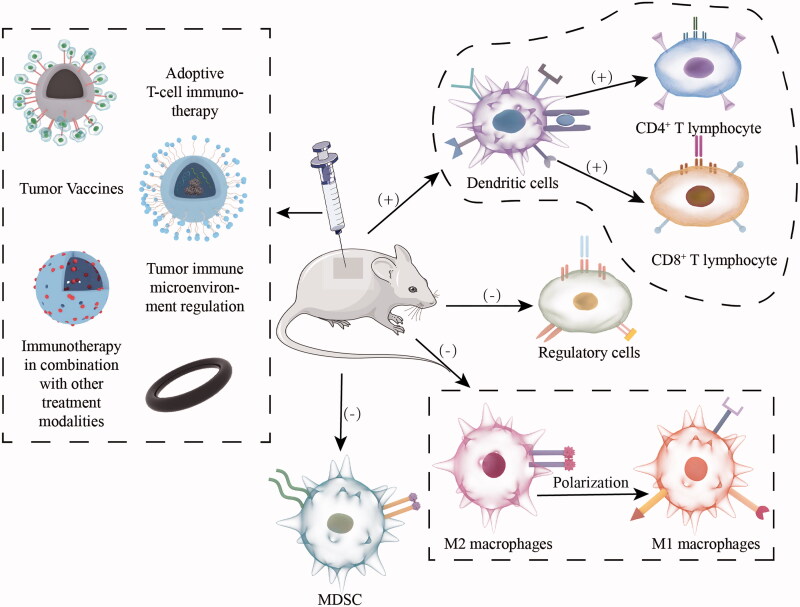
Inorganic nanomaterials involved in different types of cancer immunotherapy and their regulatory role on immune system.

## I. Inorganic nanomaterials

**Figure 2.**
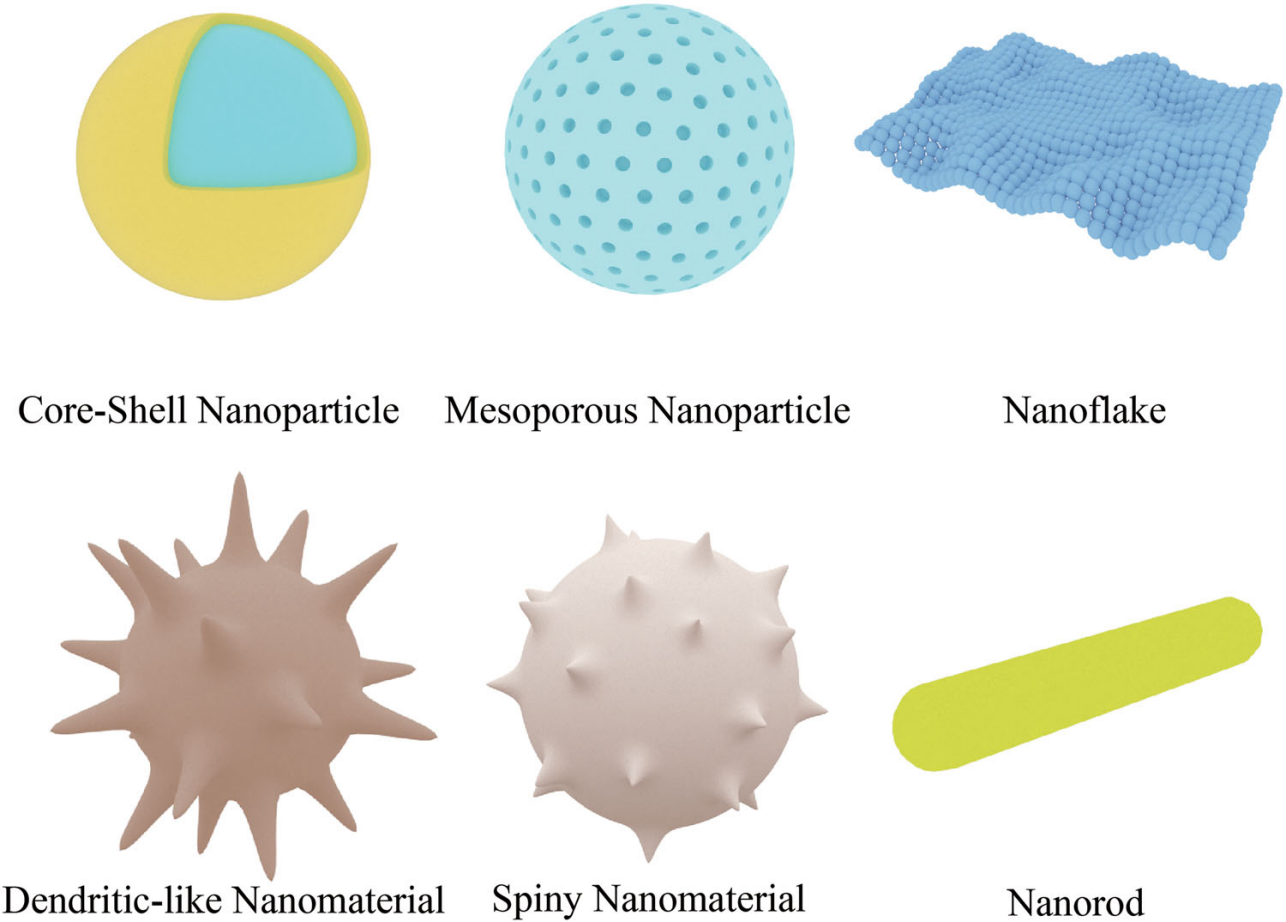
Different morphologies of inorganic nanomaterials.

According to their composition, inorganic nanomaterials can be divided into silica nanomaterials, manganese dioxide nanomaterials, gold nanomaterials, magnetic nanomaterials, etc.; according to their morphology, they can be divided into mesoporous nanoparticles, nanospheres, nanorods, spiny nanomaterials, nanoflakes, and dendritic-like nanomaterials (Hashemi et al., 2020) (Figure 2).

As an effective carrier, inorganic nanomaterials have high specific surface areas, so they can carry more immunotherapeutic drugs such as tumor antigens and immune adjuvants to the tumor site under the same circumstances, and the drug release curve shows that inorganic nanomaterials can maximize the therapeutic effect of drugs after delivering them to the tumor site with extremely high release rate (Hashemi et al., 2020). Among them, mesoporous silica has a larger specific surface area than ordinary nanomaterials due to its unique mesoporous structure, which enhances its drug-carrying capacity by incorporating therapeutic drugs into its surface mesopores (Nguyen et al., 2019). In addition, the drug loading capacity of inorganic nanomaterials can also be modulated by modifying the surface potential of the inorganic nanomaterials through the modification of groups on the surface of the inorganic nanomaterials, which in turn changes the strength of the electrostatic force between the inorganic nanomaterials and the bound drugs (Wang et al., 2021).

**Figure 3.**
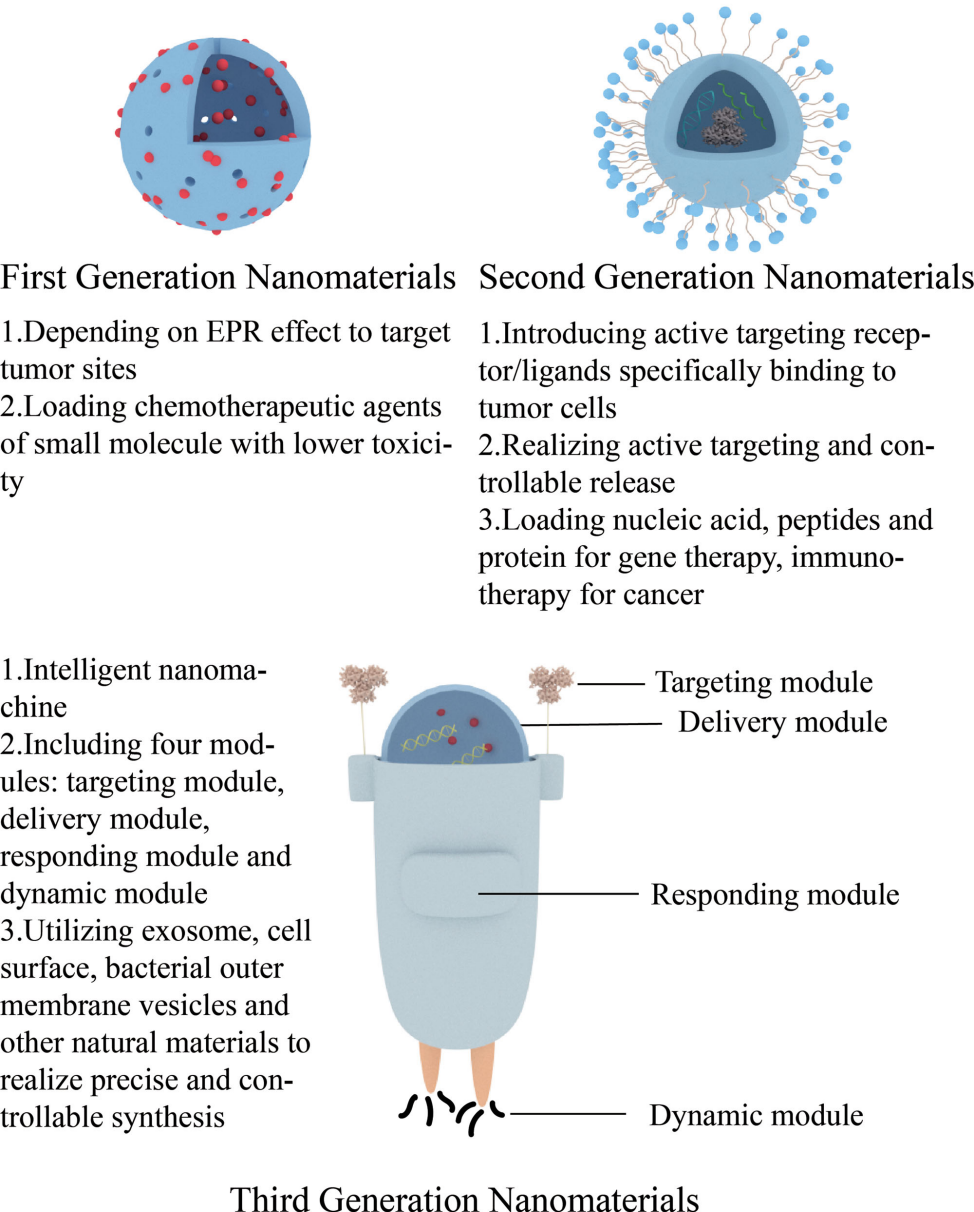
Evolution of the inorganic nanomaterials and main characteristics of each generation of nanomaterials.

Inorganic nanomaterials have good targeting properties. Studies have shown that inorganic nanomaterials can rely on the EPR effect (enhanced permeation blocking effect) to enhance their passive targeting to tumor sites. In normal tissue blood vessels, the distance between two inner cells is about 2 nm, while in tumor blood vessels, the distance between two inner cells is 100–150 nm, and the particle size of anti-tumor nanoparticles is generally 10–150 nm, which has super penetration power. It can penetrate tumor tissues and promote accumulation, and this effect is known as the EPR effect, which mediates the passive targeting ability of inorganic nanomaterials to tumor sites (Albanese et al., 2012). In addition, the active targeting effect of inorganic nanomaterials can be enhanced by integrating ligands that can be specifically recognized and conjugated to tumor cell receptors with inorganic nanomaterials (Gong et al., 2021). Inorganic nanomaterials can be classified into first-generation nanomaterials and second-generation nanomaterials according to their targeting effects. First-generation nanomaterials passively target tumor sites due to their EPR effect, while second-generation nanomaterials build on the existing advantages of nanomaterials by adding new functions, such as active targeting (Wang et al., 2021). The good targeting property of inorganic nanomaterials can induce site-specific enrichment of the carried drugs and reduce the nonspecific diffusion of drugs to other organs causing organ toxicity or systemic toxicity, and most of the inorganic nanomaterials can be excreted through the liver and kidneys after their action, and have good biocompatibility (Hashemi et al., 2020). In addition, the surface modification of inorganic nanomaterials, such as cationic coating, can increase the retention time of the drug in the blood circulation and further enhance the efficacy of the drug. Inorganic nanomaterials themselves have adjuvant properties, which can stimulate the body to produce an immune response and assist the therapeutic effect of tumor vaccines (Nguyen et al., 2019). In recent years, the third generation of nanomedicine has gradually emerged, also known as intelligent nanomachines, including natural materials such as exosomes, cell membranes, bacterial outer membrane vesicles, and microparticles for preclinical research, as well as nanomachine drugs prepared by DNA framework self-assembly, DNA origami and other precise and controllable carrier synthesis technologies, which have the characteristics of environmental responsiveness, focal active recognition, and specific response, and thus have strong clinical application value and prospects (Liang et al., 2019; Wang et al., 2020; Li et al., 2018) (Figure 3).

Most inorganic nanomaterials have good biosafety. Numerous studies have shown that most inorganic nanomaterials are mainly enriched in tumor, liver, and kidney sites after entering the body through subcutaneous or intravenous injection (Shi et al., 2020). Firstly, the high tumor-targeting property of inorganic nanomaterials can effectively reduce systemic toxicity and toxic side effects on normal organs. Secondly, inorganic nanomaterials are degraded by the mononuclear macrophage system in the liver and cleared out of the body through urine or feces after performing their function at the tumor site (Hashemi et al., 2020). In addition, the *in vivo* degradation products of most inorganic nanomaterials commonly used today are not harmful to the organism. For example, phosphate, a degradation product of inorganic black phosphorus, can further participate in the body’s calcium and phosphorus metabolism (Kuntz et al., 2017), and ferrous ions, a degradation product of magnetic nanoparticles, can also form ferritin to participate in iron metabolism (Vela, 2018). Therefore, the excellent biocompatibility and biodegradability exhibited by inorganic nanomaterials can effectively ensure their safe application in tumor immunotherapy.

Inorganic nanomaterials are characterized by easy access to raw materials, simple synthesis process, standardized operation, and high reproducibility, making it possible to realize the mass production of nanomaterials. Moreover, many inorganic nanomaterials, such as gold nanomaterials and magnetic nanoparticles, possess photothermal properties or magnetic properties, which can induce photothermal therapy by themselves or enhance tumor targeting ability by applying an external magnetic field, thus greatly enhancing the intensity of tumor immune response. Based on the above reasons, inorganic nanomaterials have received extensive attention in the field of tumor immunotherapy.

## II. Cancer immunotherapy

As an emerging tumor treatment modality, cancer immunotherapy stimulates the activation of the body’s anti-tumor immune system through indirect tumor antigen or immune adjuvant stimulation, direct adoptive transfer of immune cells or blocking immune checkpoints (Li et al., 2022) to effectively eliminate tumors, improve prognosis and prolong patients’ survival time. They are characterized by high specificity, high response intensity and rapid response, but demonstrate weak immunogenicity of tumor antigen, low tumor targeting ability, and low efficiency of in vitro immune cell expansion. However, the introduction of inorganic nanomaterials into cancer immunotherapy can effectively compensate for the above limitations; therefore, combining inorganic nanomaterials with tumor immunotherapy can significantly improve tumor treatment effects and patient prognosis.

### 1. Adoptive T-cell immunotherapy

A large number of researches have proved that the adoptive T-cell therapy (ACT, adoptive T-cell therapy), by activating and expanding autoimmune T cells in vitro and then re-infusing them back into tumor patients with appropriate cytokine stimulation, has prompted them to perform the function of killing tumor cells (Rosenberg & Restifo, 2015). The treatment has achieved remarkable results in acute B-lymphocytic leukemia, non-Hodgkin’s lymphoma, and other hematologic tumors, but its use in the treatment of solid tumors is subject to many limitations, such as the inability of therapeutic T cells to expand in vitro to generate sufficient numbers of effector cells due to insufficient stimulation signals, and the low targeting of peripatetic therapeutic T cells (Gong et al., 2021). In previous decades, a good deal of research has demonstrated that introducing inorganic nanomaterials into ACT therapy can significantly improve its therapeutic efficacy in solid tumors.

First, three signals are required for the activation of T cells in vitro, namely T cell receptor (TCR) stimulation, co-stimulatory signaling molecules (CD80/CD86), and secretion of pro-survival cytokines (IL-2), which are mostly derived from antigen-presenting cells (APCs) and activate the corresponding cell signaling pathways by interacting with T cells, thereby activating T cells. Based on this, researchers synthesized commercial microspheres (Dynabeads), which were encapsulated with anti-CD3 antibodies and anti-CD28 antibodies to provide TCR and co-stimulatory activation signals and, at the same time, adjuvantly add exogenous interleukin IL-2 to further promote the proliferation and activation of polyclonal T cells, which is one of the most commonly used in vitro activation systems. However, the limitation of this product is that it cannot mimic the presentation of the three signals by APCs in the *in vivo* environment; therefore, this method is not efficient in expanding T cells and cannot clone a large number of functionally normal T cells; in addition, because these microbeads are non-degradable, they need to be separated in advance before T cells are returned to the patient, which undoubtedly increases the economic cost and time cost of the treatment (Li & Kurlander, 2010). Autologous monocyte-derived dendritic cells (MoDC) are another common system for antigen-specific expansion of naive T cells and memory T cells, but its further clinical application is limited by its cumbersome cell manufacturing procedure, high heterogeneity among donor MoDC, and the need for routine restimulation (Wölfl & Greenberg, 2014). To overcome the shortcomings of the above two T-cell in vitro expansion activation systems, Cheung et al. (2018) discovered that the ability of MSRs (mesoporous silica micro rods) to load cytokines and form 3D scaffolds to allow them to be used for mass expansion of T cells in vitro, and developed a composite MSR-SLB formed by loading lipid bilayers (SLBs) on the surface of MSRs with high aspect ratios. SLBs are capable of presenting a combination of T cell activation signals at a predetermined density on a liquid lipid bilayer, which is used to mimic the way APC naturally triggers T cell proliferation; the mesopores of MSR can be used to load IL-2 and it can form a 3D scaffold. In T-cell culture medium, MSR-SLB can self-assemble into 3D scaffolds called APC-mimetic scaffolds (APC-Ms), which facilitate the continuous paracrine secretion of soluble signals into nearby T cells and can present surface activation signals and soluble activation signals to T cells in a similar manner to natural APC after functionalization of T-cell activation signals by SLB. When the scaffold adheres to anti-CD3 and anti-CD28 antibodies, it can expand both mouse and human polyclonal T cells; by linking peptide-loaded major histocompatibility complex (MHC) and anti-CD28 antibodies, it can induce antigen-specific expansion. MSR-SLB significantly increases the in the vitro expansion rate of murine-derived and human-derived T cells that feature antigen specificity and polyclonal binding, thus stimulating the production of large amounts of cytotoxic CD8^+^ T cells characterized by specific binding to antigen peptide and secretion of IFN-γ as well as TNF-α, greatly accelerating production speed of therapeutically competent T cells.

After passing the activated and amplified therapeutically competent T cells in vitro into patients with solid tumors, it was found that the passing T cells would disperse to multiple sites and only a few T cells reached the tumor site, showing weak tumor-targeting property. To overcome this defect, Sanz-Ortega et al. (2019) designed a magnetic targeting therapy for T cells by first synthesizing 12.5 nm diameter magnetic nanoparticles (MNP) with an iron oxide core and 3-APS coating, and attaching them to OT-I CD8^+^ T cells without affecting the antigen-specific CD8^+^ T cell anti-tumor activity, and relaying them into a homozygous mouse model. They found that a significant decrease in the percentage of OT-I CD8^+^ T cells infiltration at the tumor site and an increase in the percentage of OT-I CD8^+^ T cells infiltration in the tumor-targeted lymph nodes happened when researchers placed magnets near the tumor during relay loading of OT-I CD8^+^ T cells with APS-MNP, while no significant increase in the number of OT-I CD8^+^ T cells infiltrating into tumor sites has been observed after modifying them with APS-MNP. Thus, although the use of an external magnetic field inhibited the infiltration of relayed tumor-specific CD8^+^ T cells at the tumor site, it promoted their retention in tumor-targeting lymph nodes, which in turn improved the targeting of APS-MNPs-modified tumor-specific T cells and was effective in preventing tumor recurrence and metastasis.

### 2. Tumor vaccines

**Figure 4.**
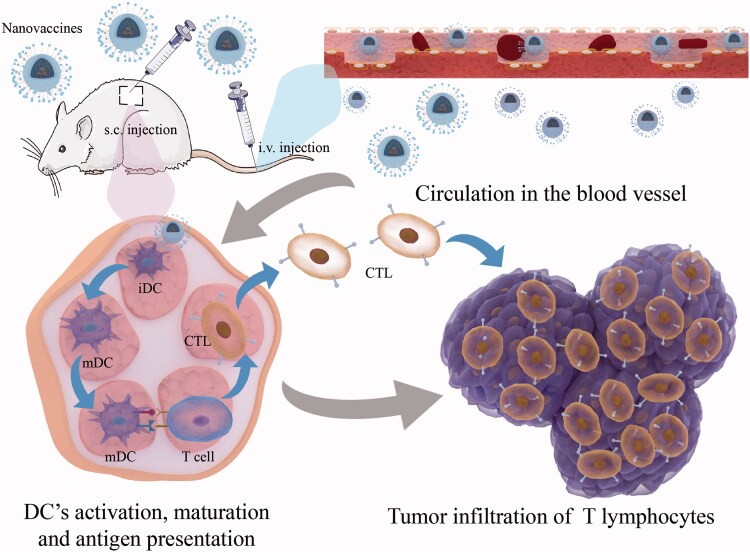
Mechanism of activation of cellular immune response by nanovaccines through Subcutaneous or intravenous injections.

As a kind of active immunity, the tumor vaccine aims to achieve tumor clearance by activating an adaptive anti-tumor immune response in the body. After tumor vaccine injection, some of the tumor-specific antigen components contained in the vaccine enter the tumor-targeting lymph nodes through blood circulation, where the antigen-presenting cells (especially the specialized antigen-presenting cells DC cells) first recognize the tumor-specific antigen, then internalize it into the cytoplasm and promote the activation and maturation of DC cells through a series of mechanisms; the internalized antigen is further processed in the intracellular bodies or lysosomes to form short peptides, which bind to major histocompatibility complex I (MHC-I) or II (MHC-II) and collaborate with co-stimulatory molecules to present antigenic information to primary T cells and induce their proliferation and activation (Hobernik & Bros, 2018) (Figure 4). Depending on the vaccine components, they can be divided into cellular tumor vaccines and antigenic tumor vaccines. The most common cellular tumor vaccines are DC cell vaccines, and antigenic tumor vaccines include tumor cell lysates, model antigens OVA, tumor-specific neoantigens, etc (Hashemi et al., 2020). In recent years, a large number of tumor heterogeneities in clinical practice have driven the introduction of personalized tumor vaccines.

Although some progress has been made in the preparation of tumor vaccines, the low immunogenicity, the activation of systemic non-antigen-specific induced by adjuvants will weaken their ability to induce a continuous and powerful anti-cancer immune response (Saxena et al., 2021; Sangro et al., 2021). To further enhance the ability of tumor vaccines to activate the body’s immune system against tumors, Qin and his colleagues (Qin et al., 2021) proposed that the shortcomings mentioned above can be compensated at the five critical cascading events: Loading tumor-specific antigens by nanoscale drug delivery systems (L); Draining tumor antigens to lymph nodes (D); Internalization by dendritic cells (DCs) (I); Maturation of DCs for costimulatory signaling (M); and Presenting tumor-peptide-major histocompatibility complexes to T cells (P) (LDIMP cascade in short). The response intensity of one or more steps in the LDIMP cascade can be improved by changing the size, morphology, charge, surface roughness, types of tumor antigens and adjuvants loaded on the inorganic nanomaterials, etc., which in turn significantly improve the therapeutic efficacy of tumor nanovaccines by mediating the enhancement of one step or several steps in LDIMP cascade (Table 1). Firstly, the adjuvant properties of inorganic nanomaterials themselves enable them to effectively assist tumor vaccine activation of T lymphocytes (Hashemi et al., 2020); secondly, some inorganic nanomaterials with special morphology (e.g. MSN) can load more tumor antigens into DC cells through their nanoscale volume and surface mesopores, thus improving the ability of DC cell activation and maturation, and antigen cross-presentation (Nguyen et al., 2019); furthermore, the surface modification of inorganic nanomaterials can regulate the loading and release of tumor antigens and immune adjuvants, which can effectively prolong their action time in the blood circulation and prevent their early release before reaching the target site (Wang et al., 2021). In addition, a special tumor microenvironment can be a prominent stimulus to achieve the specific release of tumor vaccines. The tumor environment features low pH, high-level GSH, high-level ROS, hypoxia, overexpressed enzymes, and high-level ATP. Different environmental stimuli contain corresponding sensitive groups. Inorganic nanomaterials can introduce chemical bonds or molecules with specific sensitive groups by surface modification, respond to the related environmental stimulus, and realize tumor-targeted release and accumulation, thus improving the therapeutic effect of cancer immunotherapy (Peng et al., 2022). Therefore, inorganic nanomaterials, as a successful drug delivery system, are introduced into the construction of tumor vaccines to form tumor nano vaccines, which can further enhance the strength of the body’s anti-tumor immune response and improve the therapeutic effect of tumor vaccines (Figure 4).

**Table 1.** Summary of recent research on tumor vaccines based on inorganic nanomaterials for cancer immunotherapy, including the nanomaterials involved, payloads, and the effects.

Nanomaterial type	Material properties	Payloads	Key findings	Reference
HA nanorod	Lengths: 100 nm, 200 nm, 500 nm, 1 μm, 10 μm	OVA	Influencing immune response in size-dependent manner.	(Wang et al., 2019)
MSN	Diameter: 26.7 ± 4.8 nm Pore size: 2.3 nm Surface modification: PEG, TA	CDG	Stimulated the secretion of IL-6, IL-1β, and IFN-β; Enhanced infiltration of leukocytes and dramatic tumor growth inhibition.	(Chen et al., 2020)
MSN	Diameter: 80 nm Pore size: 7.8 nm (MSN-S), 10.3 nm (MSN-M), 12.9 nm (MSN-L)	OVA	MSN-L shows: Prominent tumor growth inhibition; Enhanced OVA-specific CD4^+^, CD8^+^T lymphocyte activation and maturation.	(Hong et al., 2020)
α-Al_2_O_3_ nanoparticles	Modified with 4-hydroxybenzonic acid and coupling with ubiquitin-binding protein V × 3	Ups from 4T1 cell lysate	Enhanced DC activation, maturation and secretion of IFN-γ; Significant tumor growth inhibition and prolonged survival time.	(Huang et al., 2020)
H-MSN	Extra-large mesopores: 20–30 nm Surface modification: PEI	OVA	Enhanced activation of DCs and increased antigen-specific cytotoxic T cells; Obvious tumor growth suppression and improved survival rate.	(Lee et al., 2020)
Silica solid sphere	Diameter: 350 ± 5 nm	OVA	Improved antigen loading capacity and enhanced DCs activation, maturation and antigen presentation; Good biodegradability and targeting ability.	(Dong et al., 2020)
BPQD	High photothermal conversion efficiency; Coated with cancer cell membrane; Loaded on a thermal sensitive gel containing GM-CSF and LPS	neoantigen	Enhanced recruitment of DCs and subsequent activation and maturation; Significant clearance of tumor cells from primary and metastasis site.	(Ye et al., 2019)
MSR + MSN	MSN Diameter: 150 nm Pore size: 20–30 nm MSR Length: 86 μm Width: 14.5 μm	OVA	Enhanced recruitment of DCs into MSR and subsequent DCs’ activation, maturation and antigen presentation; Increased number of antigen- specific T lymphocytes and inhibition of tumor growth.	(Nguyen et al., 2020)
AuNP	Diameter: 5.8 ± 0.8 nm	MUC1,α-GalCer	Induced significant antibody response and MUC1-specific CTLs; 2. Significantly delayed tumor development in tumor-bearing mice model.	(Liu et al., 2021)
XL-MSNs	Diameter: 100–200 nm Pore size：25 nm Surface modification:NH_2_	OVA, CpG	Enhanced activation and antigen presentation of DCs; Increased the number of CTLs; Inducing immune memory response to inhibit tumor growth after tumor rechallenge.	(Cha et al., 2018)
Metal-doped MSNs	Diameter: 100 nm Doped with Ca, Mg, and Zn	OVA	Highest anticancer immunity and Th1 response with MSN-Zn.	(Wang et al., 2017)
Fibrous MSNs	Diameter: 100–200 nm	OVA, Poly(I:C)	Improved anti-tumor effect and functioned as joint adjuvant.	(Wang et al., 2018)
CaCO_3_	Diameter: 259 nm Surface modification: PEI	OVA, CpG	Promoted the activation of BMDCs and specific immune response of effector T lymphocytes.	(Hu et al., 2020)
MSN	Coated with PDA	OVA, ABC	Improved antigen cross-presentation ability of immune cells; Strong immune memory to inhibit tumor recurrence and metastasis	(Huang et al., 2021)

Earlier, scientists proposed whether the spike-like structures on the surfaces of viruses and bacteria were involved in the activation of the body’s intrinsic immune response, taking into account their ability to activate the body’s intrinsic immune response after the invasion and the spike-like structures on the surfaces of viruses and bacteria. Based on this, Wang et al. (2018) synthesized a spike-like TiO_2_ nanomaterial and found that its action on the cell membrane surface could generate mechanical stress, leading to caspase-1 and NLR3-dependent K^+^ efflux and inflammatory vesicle activation, and promote the maturation and activation of DC cells in concert with TLR4 pathway, thus enhancing the intensity of T-cell anti-tumor immune response and humoral immune response. This finding not only revealed the potential role of these nanostructures in activating natural immunity but also to lay the foundation for designing spiky surfaces to enhance the immunogenicity or adjuvant capacity of particle-based tumor immunotherapy and prophylactic vaccines. In addition to the ability of nanomaterial morphology to influence the immune response of the organism, it has been reported in the literature that the morphological size of inorganic nanomaterials can also have an impact on the intensity of the antitumor immune response and the targeting of the drugs carried (Wang et al., 2019). It has been shown that rod-shaped HA (hydroxyapatite) stimulates the anti-tumor immunity of the organism in a size-dependent manner. By designing rod HA of 100 nm-10 μm and dividing them into three groups according to the length: short rod HA (length between 100 and 500 nm), medium-length rod HA (length between 500 nm), and long rod HA (length between 500 nm and 10 μm), OVA-loaded rod HA was incubated with DC cells for some time and demonstrate that short rod HA could effectively promote antigen uptake and DC’s activation maturation, while it showed strong lymph node targeting ability. The long rod HA could prolong the retention time of antigen in the lymph nodes and further recruit DC cells. Medium-length rod HA combined the advantages of long rod HA and short rod HA, which was evidenced by potent lymph node targeting ability, durable retention in the lymph nodes, enhanced internalization of antigens by DCs, activated DCs, and more powerful immune response against tumor mediated by effector T lymphocytes, thus inhibiting tumor growth.

In recent years, the advantages played by inorganic nanomaterials in the body’s tumor immune response have guided scholars to further expand their synthetic component selection and synthesize more kinds of inorganic nanomaterials. It has been noted that after inoculation of a mouse ectopic colorectal tumor model with a therapeutic calcium phosphate nanovaccine functionalized with CpG and tumor model antigen, the number of tumor cytotoxic CD8^+^ T cells was significantly increased in a type I interferon (IFN-I)-dependent manner compared with systemic injection of soluble CpG and tumor model antigen, exhibiting a significant inhibition of tumor growth (Heße et al., 2019). Huang et al. (2020) developed an adjuvant-built tumor nanovaccine, using ubiquitinated protein (UPS) as the tumor antigen and covalently binding to modified α-Al_2_O_3_ nanoparticles with the help of ubiquitin-binding protein Vx3, which can achieve efficient enrichment of UPS and synthesize α-Al_2_O_3_-UPS tumor nanovaccine. Compared with the mixture of α-Al_2_O_3_ nanoparticles and UPS, α-Al_2_O_3_-UPS elevated expression of CD80, CD86, MHC-I, and MHC-II-like molecules in DCs, suggesting a stronger ability to activate DCs and induce higher interferon-γ secretion, and also showed stronger tumor growth inhibition and survival prolongation in 4T1 tumor-bearing mice. It has been suggested (Gaidzik et al., 2013) that aberrant glycosylation of the transmembrane glycoprotein MUC1 mediates tumor formation and becomes an important component of tumor-associated carbohydrate antigens (TACA); α-galactosylceramide (α-GalCer), an immune adjuvant, activates another type of T cell with antitumor effects by binding to the MHC-like molecule CD1d to form a glycolipid ligand complex—iNKT cells (Godfrey & Kronenberg, 2004), which secrete a series of Th1-type cytokines (e.g. interferon-γ, IL-2) and Th2-type cytokines (e.g. IL-4, IL-5, IL-13) upon activation to exert anti-tumor effects (Tyznik et al., 2008). Due to the difficulty of covalent reaction between hydrophobic α-GalCer and hydrophilic antigen, Liu et al. (2021) synthesized a tumor vaccine loaded with both α-GalCer and MUC1 by introducing multivalent gold nanocarriers, and studied for the first time the immune response triggered by the coupling of α-GalCer adjuvant and antigen on the surface of gold nanoparticles, and observed the effect on tumor immune response by changing the loading ratio of adjuvant to tumor antigen. The interaction between the AuNP-based vaccine-triggered antiserum and MUC1-positive-expressing MCF-7 cells was found, and the strongest binding between AuNP and MCF-7 cells was observed when MUC1:α-GalCer was 2:1, with a significant lytic effect on MCF-7 cells. In addition, splenocytes from AuNP vaccinated mice secreted higher levels of IL-4 than controls and had the highest IL-4 secretion level and IFN-γ when MUC1:α-GalCer was 2:1. By classifying the immune cell types at the tumor sites of AuNP vaccinated mice, it was found that AuNP significantly increased the percentage of CD3^+^CD8^+^ T cells, which may lead to tumor suppression by antigen-specific cytotoxic T lymphocytes. Hu et al. (2020) designed a tumor nanovaccine combined with a gene-mediated extracellular matrix scavenger as a synergistic tumor immunization strategy to eliminate tumors, using a PEI/CaCO_3_ (polyethyleneimine-coated CaCO_3_) bifunctional delivery system as a vaccine carrier that efficiently adsorbs both the antigenic ovalbumin (OVA) and adjuvant demethylated cytosine phosphoguanine (CpG) and works as the basic adjuvant to activate bone marrow-derived dendritic cells (BMDCs). The synthesized PEI/CaCO3/OVA/CpG tumor nanovaccine (NVS) achieved significant enhancement of BMDCs activation and T cell-specific responses *in vivo*, and the adjuvant nanoparticles PEG/PEI/pSpam1 (pSpam1@NPs), which highly express hyaluronidase (Haase) in tumors, degrade the physical barrier—extracellular mesenchyme (the main component is hyaluronic acid) that inhibits infiltration of tumor vaccines and activate T cells into tumor sites, thus improving the therapeutic efficacy of tumor vaccines. SiO_2_, due to its surface rich in silicone hydroxyl groups (-SiOH), is easy to modify by coupling with different functional groups, forming various functionalized surfaces to meet biological needs. Therefore, Dong et al. (2020) prepared silica solid nanospheres (SiO_2_) and covalently attached model antigen OVA to the SiO_2_ surface to form a nanovaccine (OVA@SiO_2_) and found that the combined CpG-ODN application significantly upregulated the expression of co-stimulatory molecules (CD80, CD86), DC cell chemokine CCR7 and corresponding ligands (CCL19, CCL21), which on the one hand improved the ability of DCs to target migration to lymph nodes, and on the other hand promoted DC maturation activation and antigen cross-presentation for better anti-tumor effects. In addition, based on the theory that tumor neoantigens are expressed specifically in tumor cells and are highly immunogenic, Ye and his colleagues (Ye et al., 2019) obtained tumor cell membranes containing a large number of tumor neoantigens by a series of methods using surgically removed tumor tissues and wrapped them with black phosphorus quantum dots to construct black phosphorus quantum dot nanovesicles (BPQD-CCNVs) as a personalized photothermal vaccine. Then they loaded the nanovesicles into a thermosensitive hydrogel (BPQD-CCNVs-Gel) containing GM-CSF and LPS and injected it subcutaneously into mice after surgery. It was found that under NIR irradiation, BPQD released a large amount of heat, which increased the temperature of the irradiated part and promoted the continuous release of GM-CSF and LPS inside the thermosensitive gel, thus effectively recruiting dendritic cells to capture tumor antigens, inducing the expression of co-stimulatory molecules such as CD80 and CD86 and MHC-I and MHC-II and stimulating the activation and maturation of dendritic cells. Meanwhile, *in vivo* studies have further shown that the combination of PD-1 antibody treatment can significantly enhance the clearance of tumor cells from postoperative residual foci and lung metastases by tumor-specific CD8^+^ T cells, effectively inhibiting tumor recurrence and metastasis.

Mesoporous silica nanomaterials have received wide attention from scholars as a suitable tumor vaccine carrier due to their high porosity, high biocompatibility, easy surface modification, and their adjuvant properties (Nguyen et al., 2019). c-di-GMP (CDG), a STING pathway activator, was loaded onto mesoporous silica nanoparticles by Chen et al. (Chen et al., 2020) and found that it could protect the encapsulated CDG from rapid digestion and degradation by enzymes in serum, and the passive targeting of tumor sites was achieved through the EPR effect of MSN, which effectively reduced the systemic toxic side effects of CDG alone. In addition, because of its nanoscale size, MSN facilitates the internalization of CDG by endocytosis, thus promoting the intracellular delivery of loaded CDG and the secretion of a series of pro-inflammatory cytokines by inducing the activation of the STING pathway and the maturation and activation of antigen-presenting cells, further increasing the intensity of T-cell anti-tumor immune response. By synthesizing mesoporous silica nanoparticles (MSN) of 80 nm in diameter with three different pore sizes (7.8 nm, 10.3 nm, and 12.9 nm) and loaded with OVA, Hong et al. (2020) constructed tumor vaccines OVA@MSN-S, OVA@MSN-M, and OVA@MSN-L, and investigated the effect of MSN pore size on the antigen presentation efficiency, and found that the three MSNs with different pore sizes have similar roles in mediating targeting to tumor lymph nodes, internalizing uptake by DC cells and inducing DC cell activation and maturation. However, the MSN with larger pore size showed stronger antigen cross-presentation efficiency, which in turn significantly enhanced CD4^+^ and CD8^+^ T cell activation, secreted more IFN-γ, IL-4, and TNF-α, and increased the intensity of anti-tumor immune response, thus significantly inhibiting tumor growth and improving survival in B16F10 tumor-bearing mice. In addition, OVA@MSN-L induced the strongest humoral immune response, which further enhanced the anti-tumor effect; meanwhile, because OVA@MSN-L showed a faster degradation rate, it had higher biosafety and could also promote the faster release of the drug encapsulated within it. Inspired by this study, Lee et al. (2020) synthesized hollow mesoporous silica nanoparticles (H-XL-MSN) with extra-large mesopores based on core-shell mesoporous silica nanoparticles (Mt-XL-MSN) with iron oxide nanoparticle clusters as the core in a simple one-step reaction, and by increasing the amount of iron oxide embedded in individual Mt-XL-MSN and adding methanol to decrease the polarity of the solution, more iron oxide nanoparticle clusters were induced to aggregate to form larger cores, which could be removed under acidic conditions to form larger hollow endospores and load more tumor antigens of different molecular weights. In addition, the outer layer of H-XL-MSN is coated with polyethyleneimine (PEI) as an immune adjuvant, which modulates the loading and release of tumor antigens and enhances their immunogenicity by changing the surface charge of the particles. In vitro studies showed enhanced activation and maturation of DC after treatment with PEI-coated H-XL-MSN. *In vivo* studies showed that this tumor vaccine increased the infiltration of antigen-specific cytotoxic T cells and significantly inhibited tumor growth. Cha et al. (2018) synthesized a solid, oversized mesoporous silica nanoparticle (XL-MSN), which was applied as a prophylactic cancer vaccine by delivering cancer antigens to DCs in draining lymph nodes. They found that in the case of using equal amounts of conventional MSNs with small-size pores and XL-MSN to load antigen peptides and Toll-like receptor 9(TLR9) agonists, the latter showed stronger loading capacity, which might be due to its extra-large pore size (∼25 nm) and additional surface modification. Meanwhile, taking advantage of XL-MSN’s excellent loading capacity, dosages of XL-MSN into the body to activate the host’s immune system could be minimized, thus reducing its toxicity. In vitro studies showed that DCs treated with XL-MSN-loaded antigen and TLR9 agonists exhibited the stronger ability of DCs’ activation, maturation, antigen presentation, and secretion of pro-inflammatory cytokines. *In vivo* studies demonstrated that XL-MSNs co-delivered antigen peptide and TLR9 agonists could effectively target draining lymph nodes, induce tumor infiltration of antigen-specific cytotoxic T lymphocytes (CTL), and inhibit tumor growth. In addition, because this tumor vaccine-induced a large number of memory T cells into the tumor site after injection into mice, tumor growth was significantly inhibited when the mice were attacked by the tumor again. To enhance the intensity of the anti-tumor immune response and improve the therapeutic effect of tumor vaccines, this can be achieved not only by adjusting the mesopore size of MSN but also by surface modification of MSN, changing MSN morphology, and introducing other components when synthesizing MSN. Wang et al. (2018) synthesized a stellate fibrous MSN and found that it played a role as a synergistic adjuvant when combined with OVA and poly(I: C) to construct a tumor vaccine, thus resulting in a reduced amount of poly(I: C) and lower possibilities of triggering systemic toxicity. This scholar also found that (Wang et al., 2017) adding Ca^2+^, Mg^2+^, and Zn^2+^ to the common MSN was able to improve its degradation ability while retaining its potency as a tumor vaccine carrier, probably because non-bridging oxygen (in silicate slag, the oxygen connecting single silicon) in the metal-doped MSN could promote the degradation of the silica network more rapidly than non-bridging oxygen in the pristine SiO_2_ matrix in the MSN. Nguyen et al. (2020) developed an injectable dual-scale mesoporous silica vaccine (MSN-MSR vaccine) consisting of mesoporous silica microrods (MSR) coupled with mesoporous silica nanoparticles (MSN) that induces a durable, high-intensity antitumor immune response. MSR injection formed a three-dimensional macroporous scaffold in which release of pore-loaded DC-recruiting chemokines results in a large number of DCs being recruited into the scaffold, and MSN-loaded tumor antigens with Toll-like receptor 9 agonists were present in the interstitial spaces of the particles of the MSR scaffold. Injection of MSN-MSR vaccine was able to locally enrich DC cells as well as deliver MSN co-loaded tumor antigen and TLR-9 receptor agonist to the recruited DC cells, inducing their activation and maturation, thus promoting massive infiltration of antigen-specific CD8^+^ T cells and IFN-γ^+^ CD8^+^ T cells, showing stronger tumor-suppressive effect than single MSR or MSN vaccine. Further studies revealed that long-term DC recruitment in localized areas where the nanovaccine was present was achieved due to the long-lasting DC recruitment and subsequently durable anti-tumor response mediated by the microporous scaffold.

Huang and his colleagues (Huang et al., 2021) used the MSNs to load OVA and ammonium bicarbonate (ABC), and then synthesized MSNs-ABC@PDA-OVA nano vaccines. The authors first encapsulated ABC into the pores of MSNs, then coated PDA on the surface and loaded OVA. As the research showed, when MSNs-ABC@PDA-OVA vaccine entered DCs, OVA antigens was rapidly released under NIR irradiation, while the encapsulated ABC induced activation maturation of DC cells and cross-presentation of antigens by promoting the escape of OVA from lysosomes into the cytoplasm, and activated antigen-specific cytotoxic T lymphocytes to exert anti-tumor effects. In addition, PDA exhibited good photothermal properties and NIR irradiation led to apoptosis and necrosis of tumor cells, which could effectively remove the primary tumor. *In vivo* experiments further revealed that the cure rate of melanoma after a single injection of MSNs-ABC@PDA-OVA combined with a round of PTT treatment was as high as 75%. This nanovaccine combines PTT with immunotherapy, which not only effectively suppresses the primary tumor but also creates a strong immune memory, thus effectively inhibiting tumor recurrence and metastasis.

### 3. Tumor immune microenvironment regulation

The tumor immune microenvironment has a significant position in regulating the immune response against the tumor of the organism. Although current studies show that active immunity such as tumor vaccines can induce the infiltration and activation of T cells, NK cells, DC cells, and other immune cells and enhance the intensity of immune response, the body can form a tumor immune microenvironment that negatively regulates the intensity of immune response against tumor and hinder body’s immune system’s killing effect on the tumor through multiple mechanisms during the evolution of tumors, such as secreting a large number of immunosuppressive cytokines (IL-4, IL-10, etc.) and related enzymes (indoleamine 2,3-dioxygenase, etc.), inducing a great number of immunosuppressive cells (myeloid-derived suppressor cells, M2 macrophages, tumor-associated macrophages, regulatory T cells, etc.) to infiltrate into the tumor site and so on (Rabinovich et al., 2007). Therefore, therapies with a focus on reversing the tumor immunosuppressive microenvironment have attracted widespread attention from tumor biologists. Inorganic nanomaterials combined with tumor immunosuppressive microenvironment modulators can further improve the therapeutic effect.

It has been reported in the literature (Méndez-Blanco et al., 2018) that the anti-angiogenic drug sorafenib has good therapeutic effects in advanced hepatocellular carcinoma. However, the reduction of a large number of functional blood vessels can further aggravate the hypoxia at the tumor site, inducing the formation of an immunosuppressive tumor microenvironment. Chang et al. (2020) synthesized MnSor nanomaterials, which transported MnO_2_ to the tumor site and then reacted with H_2_O_2_ to generate a large amount of oxygen, ameliorating the hypoxia at the tumor site, reduced hypoxia-induced tumor-associated macrophages (TAMs) infiltration, and reprogrammed the immunosuppressive TME. In this study, they observed an increase in numbers of immunostimulatory M1 macrophages, a decrease in numbers of immunosuppressive M2 macrophages, enhanced tumor infiltration of CD8^+^ CTL, leading to significantly improved therapeutic efficacy of whole-cell tumor vaccine. Indoleamine 2,3-dioxygenase (IDO) is a human extrahepatic tryptophan metabolism rate-limiting enzyme that mediates tryptophan (TRP) depletion and limits TRP availability in tumor cells and innate immune cells, thus triggering an effector pathway that interferes with cytotoxic T cell activation while inducing proliferative activation of regulatory T cells (Tregs) (Löb et al., 2009). Lu et al. (2017) formed OX/IND-MSNP by coupling IDO inhibitor and oxime (IND) to phospholipids, self-assembling them into nanomicrocapsules or doping into lipid bilayers encapsulating mesoporous silica nanoparticles (MSNP), and internally loading ICD-inducing chemotherapeutic drug oxaliplatin (OX). After intravenous injection, OX/IND-MSNP was first passively targeted to the primary tumor via EPR effect, suppressing the formation of the immunosuppressive microenvironment of the tumor by inhibiting IDO metabolic pathway and downregulating the level of Foxp3^+^ T cells (Treg cells) mediated by IND, which plays a positive role in the treatment of pancreatic ductal adenocarcinoma. Cyclic GMP-AMP synthase(cGAS)/stimulator of interferon genes(STING) pathway works as a significant regulator in inherent immune response, and cytosolic DNA is an important initial factor to activate this pathway (Dou et al., 2017). Mn^2+^ can also be an effective initiator to enhance the activation of cGAS and STING (Wang et al., 2018). In addition, a piece of the literature showed that aggressive cancer cells expressed more phospholipase D than normal cells, which could be involved in reactions of phospholipid(PL) hydrolysis (Scott et al., 2009). Based on these backgrounds, Hou et al. (2020) successfully synthesized amorphous manganese phosphate(APMP)-DOX NPs coated with phospholipid(PL). When PL/APMP-DOX were uptake into tumor cells, the PL shell could be degraded by phospholipase. Then in the acidic environment, APMP-DOX quickly released Mn^2+^ ions (Hao et al., 2017) and DOX targeting DNA damage. At last, released Mn^2+^ ions and cytosolic damaged DNA collectively activated the cGAS/STING pathway, promoting the secretion of Type I IFN, enhancing the cytotoxic activity of CTL and NK cells, and demonstrating a strong anti-tumor immune response to inhibit tumor growth. In addition, Zhao et al. (2022) introduced a new type of yolk-shell nanohybrids after structural and morphological optimization——Fe_3_O_4_@C/MnO_2_-PEGA(FCMP) and utilized its innate immune regulatory effects to reprogram macrophages into M1 phenotype, promoting DCs maturation and regulating immunosuppressive TME, thus improving anti-cancer treatment efficacy.

### 4. Immunotherapy in combination with other treatment modalities

In addition to the cancer immunotherapy mode mentioned above, immune checkpoint blockade (ICB)has enjoyed wide popularity in the field of cancer immunotherapy during previous decades. Common immune checkpoints include PD-1(programmed cell death protein 1), PD-L1(programmed cell death protein 1 ligand), and CTLA-4(cytotoxic T-lymphocyte-associated protein 4), which are often expressed on the surface of T lymphocytes, cancer cells, DCs(dendric cells), Treg(regulatory T lymphocyte), NK cell(Natural Killer cells) and so on (Sangro et al., 2021). When these immune checkpoints interact with their corresponding receptors/ligands, immune cells involved will inactivate or exhaust, hindering their tumor suppressive ability. Based on this, researchers have developed various monoclonal antibodies targeting PD-1, PD-L1, or CTLA-4 to block the interaction. Among them, monoclonal antibodies against PD-1 and PD-L1 have demonstrated good treatment effects against various cancer types (Sullivan et al., 2019). However, the further clinical application has been hindered due to its low response rate in cancer patients(10–30%), which may depend on the cancer type (Kalbasi & Ribas, 2020). Research demonstrates that cancer can be classified into “hot” tumors and “cold” tumors according to T lymphocytes infiltration into the tumor site and immunogenicity of the tumor itself, and most patients bear the “cold” tumor with less T lymphocyte infiltration and immunogenicity, thus inadequately responding to ICB treatment (Wang et al., 2018). To solve this issue, reversing the “cold” tumor into a “hot” one to enhance its sensitivity to ICB treatment plays a significant role. One of the effective means to achieve such reprogramming is to induce immunogenic cell death (ICD), which brings about a series of changes in cell surface proteins and secreted soluble mediators (Siegel et al., 2019): tumor-associated antigens (TAAs) released from dying tumor cells can enhance the uptake of tumor antigens by DCs, promote DCs’ activation and maturation, and subsequently improve T cell’s activation and proliferation, thus inducing strong immune responses against tumor (Wang et al., 2021); tumor cells secrete large amounts of ATP, which has the effect of inducing myeloid cells to differentiate into antigen-presenting cells (Heath & Carbone, 2001); tumor cells release large amounts of high mobility group protein 1 (HMGB-1), which binds to DC surface receptors and promotes DC maturation and exhibits immune adjuvant-like effect (Gong et al., 2021); dying tumor cells express high levels of calreticulin (CRT) on the cell membrane, which sends “eat me” signals to DC cells and promotes antigen uptake by DCs through surface receptors. These changes and mediators make tumor cells more sensitive to immunotherapy (Yang et al., 2020).

Numerous studies show that chemotherapy(CT), photothermal therapy(PTT), photodynamic therapy(PDT) and magnetic hyperthermia therapy(MHT) can effectively induce immunogenic cell death, increase the immunogenicity of tumor cells and enhance the antigen-presenting ability of immune cells, thus activating a large number of T cells to infiltrate into the tumor site and inhibiting tumor growth at the primary site. When combining them with ICB therapy, the metastasis and recurrence of tumors could be remarkably inhibited (Zhang et al., 2020) (Table 2).

**Table 2.** Summary of recent research on combination of other therapeutic modalities based on inorganic nanomaterials with cancer immunotherapy, including inorganic nanomaterials involvement in CT, PTT, MHT, MTD, PDT with ICB.

Nanomaterial Type	Material properties	Payload	Modality of ICD induction	Key findings	Reference
dHMLB	Diameter: 186.4 ± 5.0 nm; Pore size:5 nm	ATRA, DOX, and IL-2	CT	Activating tumor infiltrating T lymphocytes and NK cells; Promoting the secretion of IFN-γ and IL-12; Negatively regulating MDSCs; Apparently inhibiting tumor growth and metastasis.	(Kong et al., 2017)
MSN	Diameter: 82–83 nm	OXP	CT	Enhanced infiltration of DCs and CTLs into tumor sites; Improved tumor-killing ability of CTLs.	(Lu et al., 2017)
MSN	Functionalized with benzaldehyde ; Modified with PDA, PEG and FA	P-gp siRNA, DOX	CT, combined with PTT and gene therapy	pH-responsive release of DOX Controllable release of P-gp siRNA by the regulation of “DOX pore lid”.	(Cheng et al., 2017)
Gold nanoparticles	Modified with legumain proteins binding target	DOX, HCQ	CT, PTT	Passively targeting glioma sites; HCQ inhibited DOX- induced cytoprotective autophagy and tumor angiogenesis mimicry; Effectively prevent tumor recurrence.	(Ruan et al., 2019)
Fe_3_O_4_ superparticle	Diameter: 149.8 nm; Surface modification: PLGA; Magenetism	R837	PTT combined with PD-L1 antibody	Higher targeting ability under the assistance of magnet at tumor site; Stronger anti-tumor immune response; Significant inhibition of primary tumor and tumor’s lung and liver metastases when combining with PD-L1 antibody.	(Ge et al., 2018)
Fe_3_O_4_ magnetic nanoparticle	Coated with DPA-PEG	R837, ICG	PTT, combined with immunotherapy	Prolonged circulating action time *in vivo*; Improved tumor targeting ability under the application of external magnetic fields; Increased intensity of anti-tumor immune response and significant inhibition of tumor growth, recurrence and metastasis.	(Fan et al., 2020)
BPQD	Coated with RBC cell membrane	/	PTT	Prolonged circulating and retention time at the tumor site; Improved tumor infiltration of CD8^+^T cells and significant inhibition of tumor growth at primary and metastasis site when combining with PD-1 antibody.	(Liang et al., 2019)
BPNSs	Coated with PDA; Modified with NH_2_-PEG-Apt;	DOX, P-gp siRNA	PTT, CT and gene therapy	Active targeting to tumor sites; Improved stability of BPNSs due to the PDA coating; Effective stimulation of anti-tumor immune system by ICD and decreased expression level of P-gp.	(Zeng et al., 2018)
Gold nanorods	High photothermal conversion efficiency	CRISPR/Cas9 plasmid	PTT and PD-L1 genome editing techniques	Transferred the “cold” tumor to “hot” one; Inhibited primary tumor growth and metastasis; Formation of immune memory and prevention of tumor recurrence.	(Tang et al., 2021)
HAuNS	High photothermal conductivity	CpG, AUNP12	PTT combined with PD-1 blocking peptide	Promoted antigen presentation of DCs and activated T lymphocyte, NK cells; Transformed sustained release of AUNP12 into triggered release.	(Luo et al., 2018)
Ag_2_S-PAsp-cRGD	Ag_2_S: High photothermal conversion efficacy	DOX	PTT combined with CT	Enhanced antigen presentation of DCs and differentiationof T cells	(Han et al., 2022)
Superparamagnetic CoFe_2_O_4_@MnFe_2_O_4_ nanoparticles	Relatively high saturation magnetization	/	MHT combined with checkpoint blockade immunotherapy	Promoted activation of DCs and CTLs; Combined therapy inhibited primary and metastatic tumor growth.	(Pan et al., 2020)
ZCMF	Core-shell structure; Sequestered by the mononuclear phagocyte system, especially in liver	/	MHT	Completely suppressed xenograft and orthotopic liver tumor growth via induced NK-cell antitumor immunity.	(Pan et al., 2021)
FeNPs	High magnetic saturation intensity; Generate sufficient heating under a low-power AMF	/	MHT combined with CTLA-4 antibody	Effective tumor ablation to induce ICD; Inhibited tumor metastasis and strong immune memory effect *in vivo*.	(Chao et al., 2019)
FVIO	Modified with mPEG and NH_2_; efficient heat induction; good suspension and fast magnetic response	/	MHT combined with PD-L1 checkpoint blockade	Inhibited the immunosuppressive response and increased CD8^+^ CTLs infiltration; 2. Suppressed metastatic spreading and distant growth of tumors.	(Liu et al., 2019)
FVIOs-GO	FVIO: high magneto-thermal conversion efficiency;GO: good electronic and thermal conductivity and ability to produce ROS	/	MTD	Provoked a strong immune response at a physiological tolerable temperature; Promoted macrophage polarization to M1 phenotype and T lymphocyte infiltration.	(Liu et al., 2020)
UCMSs	Diameter: no less than 100 nm; Further enlarged pore size;	MC540, OVA, CT26 tumor cell fragments	PDT	Improved loading efficiency of antigen and MC540; Enhanced anti-tumor immune response.	(Ding et al., 2018)
CAGE	Hypoxia responsive; PEG modified; Ce6-doped MSNs	CpG	PDT	Improved anti-tumor immune response intensity under the hypoxia tumor microenvironment.	(Im et al., 2019)
BPNSs	High photodynamic conversion ability; II-type heterojunction to enhance the therepeutic effect of PDT	/	PDT	Oxygen synthesized by photosynthesis of Ch1 cells can alleviate the effect of tumor hypoxia on PDT therapy; The II-type heterojunctions formed between BPNSs and chlorophyll in Ch1 cells induced the production of more reactive oxygen species; Enhanced anti-tumor immune response due to PTT-induced ICD and immunostimulatory effect of Ch1 cells.	(Ou et al., 2022)
Gold nanoparticles	Diameter:150 nm Encapsulated with HA; Surrounded by RBC membrane at the outermost layer	PXTK, PheoA	PDT	Particles with 150 nm as its size possess the best tumor penetration and retention ability; Improved anti-tumor immune response induced by ICD under the effect of ROS; A positive feedback was formulated by the production of ROS from mitochondria induced by the byproduct of PXTK and the facilitated ROS-responsive hydrolysis of PXTK.	(Yu et al., 2019)

#### 4.1. Chemotherapy-enhanced immunotherapy

Chemotherapeutic drugs such as doxorubicin (DOX) and oxaliplatin(OXP) exert great tumor cell killing effects and induce ICD at the tumor site. Mesoporous silica nanoparticles(MSNP) containing a large number of mesoporous can load more chemotherapeutic drugs and induce more ICD, enhancing anti-tumor immune response intensity. Meanwhile, MSPs possess good targeting ability can reduce potential systemic toxicity caused by chemotherapy. Kong et al. (2017) constructed a lipid-coated biodegradable hollow mesoporous silica nanoparticles (MLB), which is co-encapsulated with all-trans retinoic acid (ATRA), doxorubicin (DOX), and interleukin-2 (IL-2) for chemoimmunotherapy to promote the tumor infiltration of active T lymphocytes and NK cells, and subsequent secretion of cytokines IFN-γ and IL-12, thus exerting better anti-tumor effects. By loading oxaliplatin (OXP) inside mesoporous silica nanoparticles (MSNP), Lu et al. (2017) found that it could inhibit primary PDAC (pancreatic ductal adenocarcinoma) by inducing ICD, thereby promoting massive infiltration of DCs and cytotoxic T cells into the tumor microenvironment, leading to enhanced phagocytosis of tumor antigens by DCs, and thus enhancing the tumor-killing ability of cytotoxic T cells. Cheng and his colleagues (2017) used benzaldehyde functionalized post-mesoporous silica nanoparticles as the core to encapsulate P-gp siRNA within the pore to target and down-regulate the expression levels of permeable glycoproteins that mediate drug resistance in tumor cells. In order to prevent premature leakage of siRNA from the pore, MSN loaded DOX via a pH-responsive benzoic acid-imide bond and acted as “lid” to cover the pore. In addition, they also encapsulated PDA on the outermost layer of MSN, attached folic acid on its surface with its strong adhesion ability to induce active targeting of MSN to tumor cells, made additional surface loading of DOX on the surface through π-π stacking and hydrogen bonding and then synthesized M-R@D-PDA-PEG-FA-D. This study showed that after tumor cells internalized M-R@D-PDA-PEG-FA-D, PDA absorbed light energy and converted them inro hear energy under the NIR irradiation due to its high photothermal conversion ability, promoting the release of DOX from the outermost layer and apoptosis of tumor cells; meanwhile, the acidic environment of the endosome induced the breakage of the benzoic acid-imine bond connecting DOX and MSN, leading the releasing of DOX from the surface of MSN and an open state of the pore; then the internal P-gp siRNA was released into the cytoplasm for targeted degradation. This “DOX pore lid” design of nanoparticles cleverly prevented the degradation caused by the advanced leakage of siRNA and integrated gene therapy, chemotherapy, and PDA-mediated photothermal therapy into the same nano-drug delivery system, which can effectively treat multi-drug resistant tumors. Ruan and his colleagues (2019) synthesized two gold nanoparticles targeting legumain proteins within glioma cell lysosomes and loaded them with DOX and HCQ (D&H-A-A&C), which were passively targeted to glioma sites and formed aggregates in situ, leading to massive accumulation of loaded DOX and HCQ at tumor sites. It was found that HCQ was able to inhibit DOX-induced cytoprotective autophagy, thus making glioma cells sensitive to DOX again, and that inhibition of autophagy also inhibited autophagy-associated tumor angiogenesis mimicry in glioma stem cells and suppressed blood supply to the tumor. *In vivo* experiments further confirmed that D&H-A-A&C had significant anti-glioma effects, and the combination of anti-PD-L1 antibodies could alleviate the immunosuppressive microenvironment of glioma, increase the intensity of anti-tumor immune response, and the induced immune memory effect could also effectively prevent tumor recurrence.

#### 4.2. Photothermal therapy-enhanced immunotherapy

Photothermal therapy (PTT) is a noninvasive, spatiotemporally specific, and reversible treatment to induce ICD. It requires the involvement of laser irradiation and materials with high photothermal conversion efficiency. Under laser irradiation, the selected material can absorb the light of the appropriate wavelength and transform the optical energy into heat energy, which exerts significant ability in killing tumor cells and subsequently induce ICD. However, the low photothermal conversion efficiency of the current materials and inability to penetrate deep tissues limits its further application in clinical cancer treatment. Inorganic nanomaterials like gold nanorods or nanoparticles, Fe_3_O_4_ nanoparticles and black phosphorus nanomaterials have high photothermal conversion efficiency, precise tumor targeting ability, and deep tissue penetration. Ge et al. (2018) designed a Fe_3_O_4_ superparticle encapsulated with PLGA and loading immunoadjuvant R837(Fe_3_O_4_-R837SPs). By placing a magnet at the tumor site, large amounts of Fe_3_O_4_ superparticle after intravenous injection aggregate at the tumor site in the 4T1 breast cancer-bearing mice. Furthermore, after local laser irradiation of the tumor site, 4T1 breast cancer cells were vastly killed and released amounts of TAAs; Meanwhile, PTT also induced the release of R837, which was TLR7 agonist and stimulated DC’s activation and maturation. As a result, enhanced anti-tumor immune response intensity was achieved under the combined action of Fe_3_O_4_ superparticle and R837. The research further combined PTT mediated by Fe_3_O_4_-R837SPs with ICB mediated by PD-L1 antibody on 4T1 breast cancer-bearing mice and found that the combined therapy not only eradicated the primary tumor but also inhibited the tumor’s lung and liver metastases by promoting CD45^+^ leukocyte infiltration into the distant tumor site. Zhang and his colleagues (2020) constructed an ICG-loaded magnetic nanoparticles delivery system with R837 polyphenol coating. They utilized Fe_3_O_4_ magnetic nanoparticles as the core, loaded the photosensitizer indocyanine green (ICG) on the surface by electrostatic action, coated surface with DPA-PEG and loaded the immunostimulant R837 on the coating. The study showed that the coating of polyphenols could remarkably prolong the circulating action time *in vivo* and prevent its premature degradation; in addition, under the application of an external magnetic field, the nanoparticles could effectively target to the tumor site, and under the NIR irradiation, ICG rapidly converted light energy into heat energy to induce immunogenic death of tumor cells and release tumor-associated antigens to activate the body’s anti-tumor immune response. With the assistance of R837, the intensity of anti-tumor immune response was further increased, which significantly inhibited the growth, metastasis, and recurrence of tumors. It has been reported that PTT-induced temperature increase upregulated the expression of PD-L1 and further promote the formation of the immunosuppressive tumor microenvironment; therefore, the efficacy of combining PTT and ICB mediated by PD-L1 antibody was significantly reduced. To solve this problem, CRISPR/Cas9 based genome editing techniques targeting the PD-L1 genome were developed. To maximize the therapeutic effect of CRISPR/Cas9 based genome editing techniques targeting the PD-L1 genome, a proper delivery system with high transfection efficiency needs to be selected. Tang et al. (2021) chose gold nanorods as a carrier. On the one hand, gold nanorods with high photothermal conversion efficiency induced strong PTT and raised the temperature to 42 °C, which was the optimal temperature for Cas9 delivery into cells and transcription action, thus improving the gene-editing efficiency; on the other hand, the optimal temperature induced by gold nanorods-mediated PTT could promote the formation of immune memory, activate T cells, enhance T cells to infiltrate into the tumor site, transferring the “cold” tumor to “hot” tumor and improving tumor’s response to PD-L1 mediated ICB. Luo et al. (2018) utilized the high photothermal conversion efficiency of gold nanomaterials and developed hollow gold nanoshell (HAuNS) loading immunoadjuvant CpG. Under the NIR region irradiation, HAuNS demonstrated high photothermal conductivity, induced highly efficient PTT, upregulated the expression of HSP 70, and increased the ability of cytotoxic T cells to target and kill tumor cells. Moreover, the introduction of immunoadjuvant CpG could be recognized by TLR9 in DCs and promote their antigen presentation, further activating T lymphocyte, NK cells, and immune response. Meanwhile, the author encapsulated PD-1 blocking peptide AUNP12 into HAuNS and transient hyperpyrexia induced by PTT was able to realize the triggered release of AUNP12 instead of sustained release. Apart from mentioned above, Han et al. (2022) utilized high photothermal conversion efficacy of Ag_2_S (48.34%), constructed a nanoplatform named Ag_2_S-PAsp-cRGD to induce effective PTT and subsequent ICD, and demonstrated prominent anticancer effect on primary tumor and metastatic tumor, which was evidenced by improved DCs antigen presentation and enhanced proliferation and differentiation of T cells. Liang and his colleagues (2019) synthesized a nanovesicle (BPQD-RMNV). The interior is bionic black phosphorus quantum dots, whose efficient photothermal conversion efficiency mediates the onset of tumor PTT and induces immunogenic death of tumor cells; the exterior encapsulates the red blood cell membrane to prolong its retention time in circulation and tumor sites. Under the NIR irradiation, black phosphorus quantum dots absorbed light energy and generated a great deal of heat, in situ inducing apoptosis and necrosis of breast cancer cells and releasing tumor-associated antigens (TAA). Then DCs were recruited to the tumor site to capture the antigen and activate the immune system to eliminate the residual and metastatic tumor cells. *In vivo* experiments further showed that BPQD-RMNV-mediated PTT combined with anti-PD-1 antibody treatment significantly promoted the infiltration of CD8^+^ T cells into tumor sites, inhibited CD8^+^ T cell depletion at tumor sites, increased the anti-tumor activity of CD8^+^ T cells, and significantly inhibited tumor cell growth at residual and metastatic tumor sites. However, the easy reaction of black phosphorus with water and oxygen leads to structural and compositional changes, which has an impact on its photothermal efficacy and drug delivery capacity (Zhao et al., 2016) and limit the application of black phosphorus nanosheets as a multifunctional drug delivery system. To solve this problem, Zeng and his colleagues (2018) firstly synthesized black phosphorus nanosheets by liquid-phase exfoliation technique and loaded DOX and P-gp siRNA on the surface; then they coated the nanosheets with highly biocompatible polydopamine (PDA) to maintain their structural and functional stability, modified NH_2_-PEG-Apt on the surface to achieve active drug delivery to tumor cells and constructed a kind of multifunctional co-delivery drug system (BP-R-D@PDA-PEG-Apt). It was found that after the binding between NH_2_-PEG-Apt and its target on the surface of tumor cells, the black phosphorus nanosheets entered the intracellular compartment through the endocytosis of tumor cells. The acidic environment of the endosome caused the degradation of PDA on the surface and exposed the black phosphorus nanosheets; under NIR irradiation, the black phosphorus nanosheets rapidly achieved photothermal conversion, increased the local temperature, and promoted the release of DOX and P-gp siRNA, inducing immunogenic death of tumor cells and inhibiting the expression of permeable glycoproteins. Therefore, the activation of body’s immune system to exert anti-tumor effect was effectively stimulated and the ineffectiveness of chemotherapy caused by the overexpression of cell membrane permeability glycoprotein in multidrug-resistant tumor patients was well solved, realizing the synergistic application of tumor-targeted chemotherapy, gene therapy and photothermal therapy (PTT).

#### 4.3. Magnetic hyperthermia therapy-enhanced immunotherapy

Magnetic hyperthermia therapy(MHT) is another effective way to induce ICD. It demonstrates numerous advantages like low cost, noninvasiveness, high tissue penetration ability, and minimizing damage to normal tissues (Kuboyabu et al., 2016). Magnetic nanoparticles and an alternating magnetic field(AMF) are required to implement effective MHT; by applying an AMF at the tumor site, magnetic nanoparticles can produce large amounts of heat locally to destroy tumor cells (Gupta & Gupta, 2005). The release of tumor-associated antigens may induce immune responses. Pan et al. (2020) constructed superparamagnetic CoFe_2_O_4_@MnFe_2_O_4_ nanoparticles with good colloidal stability and found that it also showed relatively high saturation magnetization and excellent magnetic hyperthermia performance, resulting in effective cancer cell killing after MHT. The induced ICD could promote activation and proliferation of DCs and CTL to induce a potent immune response against distant mimetic metastatic tumors in tumor-bearing mice. Furthermore, they found that tumor growth was significantly inhibited at both primary tumor lesion and metastasis lesion when combining MHT with ICB. In 2021, Pan et al. (2021) found that most magnetic nanoparticles injected intravenously were segregated by the mononuclear phagocyte system, especially in the liver, and limited magneto-thermal efficiency. Inspired by this phenomenon, the author synthesized Zn-CoFe_2_O_4_@Zn-MnFe_2_O_4_ superparamagnetic nanoparticles (ZCMF), which have a core-shell structure, outstanding and highly manageable magnetic hyperthermia performance, and then employed ZCMF to treat orthotopic liver cancer for the first time. After the treatment of ZCMF-mediated mild MHT, liver cancer cell HepG2 presented weaker proliferative ability and stronger apoptosis ability mediated by inhibited expression of HSP-70, Cyclin D1, and upregulated expression of Bcl-2. Meanwhile, ZCMF-mediated mild MHT plays an important role in effectively activating NK cells by drastically prompting HepG2 to express more UL16-binding proteins (ULBPs) on its cell surface, which can specifically bind to natural killer group 2 member D(NKG2D) on the surface of NK cells; therefore, both xenograft and orthotopic liver tumors were almost eliminated *in vivo* due to strong immune response mediated by NK cells under mild MHT. Chao et al. (2019) utilized the high magnetic saturation intensity of pure iron nanoparticles (FeNPs) and found that it could develop large amounts of heat under a relatively low-power AMF, thus turning it into a super-effective MHT agent and resulting ineffectual tumor ablation to induce ICD. Moreover, combining regional injection of FeNPs and systemic injection of CTLA-4 antibody could induce a systemic immune response to suppress tumor metastasis and strong immunological memory to avoid tumor recurrence. Liu et al. (2019) introduced ferrimagnetic vortex-domain iron oxide nanoring(FVIO) mediated mild magnetic hyperthermia and induced an efficient immunogenic cell death evidenced by upregulated CRT expression on the 4T1 breast cancer cells, more cancer cells being uptake by phagocytes, and increased polarization of macrophages, leading to more CD8^+^ CTL infiltrating into primary and distant tumor lesions. Meanwhile, the author combined mild MHT with PD-L1 checkpoint blockade and found that the combination obviously inhibited the immunosuppressive response of the tumor by significantly down-regulating MDSCs and increasing CD8^+^ cytotoxic T lymphocyte infiltration, resulting dramatically inhibited metastatic spreading and distant growth of tumors. In 2020, the author (Liu et al., 2020) found that magnetothermodynamic(MTD) therapy is a powerful systemic cancer therapy by integrating the magnetothermal effect with the reactive oxygen species(ROS)-related immunologic effect. Based on this, he constructed FVIOs-GO nanoplatform by integrating the high magneto-thermal conversion efficiency of FVIO, the good electronic and thermal conductivity of graphene oxide (GO), and the ability of GO to produce ROS. In this experiment he also demonstrated that amplified ROS generation provoked a robust immune response at a physiological tolerable temperature below 40 °C in a hypoxic tumor microenvironment, promoting the CRT expression on 4T1 breast cancer cells, macrophage polarization to immunostimulatory M1 phenotypes, and T lymphocyte infiltration in tumors. Because of the duple influence of magnetothermal effect and ROS-related immunologic effect, the dosage of Fe and the frequency of AMF treatment were drastically reduced as well as made up for the limitation that traditional MTT only relied on the thermal effect of MNPs.

#### 4.4. Photodynamic therapy-enhanced immunotherapy

Photodynamic therapy(PDT) utilizes the photosensitizers(PS) involved in photochemical reactions to generate cytotoxic reactive oxygen species(ROS), which can not only kill tumor cells but also enhance the immune response by producing TAAs (Wang et al., 2019). PDT exhibits significantly effective results in eradicating superficial tumors, but the selected wavelength of light is often unable to penetrate deep tumor tissues, limiting its efficacy in eliminating deep tumors. A study shows that the combination of lanthanide ion-doped upconversion nanoparticles (UCNPs) and organic PS for near-infrared (NIR) light-mediated PDT within the “biological window” has a weak autofluorescence background(low interference) with strong deep tissue penetration can solve this problem (Xu et al., 2017; Chen et al., 2018). To improve the loading efficiency of organic PS and achieve deep tissue penetration, Ding et al. (2018) synthesized the monodisperse macroporous mesoporous silica-encapsulated upconversion nanoparticles (UCMSs) with a particle size of more than 100 nm, and it demonstrated good safety and biocompatibility. Meanwhile, an appropriate particle size of no less than 100 nm facilitated intracellular uptake and delivery of the loaded “cargo”. Moreover, compared with conventional mesoporous silica nanomaterials, the further enlarged pore size could significantly improve the loading efficiency of MC540, further promoting the production of singlet oxygen and enhancing the therapeutic effect of photodynamic therapy. And the enlarged mesopore improved the loading efficiency of antigen with large molecular weight. Based on this, the author formulated the UCMS-MC540-TF nanovaccine by mixing MC540 and mice CT26 tumor cell lysates and found that it was able to not only combine PDT with immunotherapy, but also achieve activation of anti-tumor immune response with the adjuvant effect of PDT-induced ICD and mesoporous silica. PS and immunoadjuvant CpG are necessary for PDT-induced ICD. The development of a nanoparticle-based delivery system (NPDS) can achieve co-delivery of PS and CpG and high tumor-targeting ability (Fan & Moon, 2015). The stability of NPDS loading PS and CpG in the cyclicprocess is important to ensure their functions; therefore, ideal NPDS possessed the ability to protect PS and adjuvant from excretion and degradation. PEG modification can stabilize NPs in the blood -streaming environment, but the modification also inhibits the interaction between NPs and tumor cells (Sun et al., 2017). To overcome this obstacle, Im et al. (2019) synthesized a hypoxia-responsive PS/adjuvant co-delivery system based on the characteristic of the tumor microenvironment, be Ce6-doped-azobenzene-glycol chitosan(GC)-PEG mesoporous silica nanoparticles(CAGE). In the case of intrinsic tumor hypoxia (oxygen content less than 2%) or sudden local oxygen depletion caused by photodynamic effects, azobenzene linker was cleaved, and PEG, CpG/GC complex shed from CAGE and Ce6-doped MSNs remained intact. Then Ce6-doped MSNs induced PDT under laser irradiation and subsequent ICD, improving anti-tumor immune response intensity with the assistance of released CpG. The efficient photodynamic effects of black phosphorus nanosheets (BPNSs)enable their application in photodynamic therapy for tumors, but the hypoxic tumor microenvironment limits the anti-tumor effects of PDT. Based on this, Ou and his colleagues (2022) proposed to use oxygen synthesized by Chlorophyceae (Chl) cells’ photosynthesis to alleviate tumor hypoxia. Then they utilized polyaspartic acid (PASP) around BPNSs to adsorb BPNSs on the surface of Ch1 cells and formed type II heterojunctions between BPNSs and the chlorophyll of Ch1 cells, which was a structure that could thoroughly separate photo-excited electrons and holes for ^1^O_2_ generation and O_2_ evolution, thus improving the conversion efficiency of light energy, generating more reactive oxygen clusters to induce immunogenic death of tumor cells, and activating the immune system of the body to exert anti-tumor effects. Meanwhile, it had been shown that the intracellular primordial polysaccharide of Chl could effectively stimulate the activation and maturation of DC cells as an immunostimulant (Chou et al., 2012), promote the infiltration of immune cells into the spleen, lymph nodes, and tumor sites, and secrete a large amount of interferon to further enhance the intensity of the body’s anti-tumor cellular immune response. Yu and his co-workers (2019) synthesized HA-encapsulated 150 nm diameter mimetic gold nanoparticles (mCAuNCs@HA) coated with erythrocyte membrane and loaded with both the photosensitizer PheoA and the ROS-responsive paclitaxel dimer precursor drug (PXTK). It was found that CD47 on the surface of erythrocyte membranes could inhibit the phagocytosis of nanoparticles by the mononuclear macrophage system (MPS) and effectively prolong their half-life in the circulation. Besides, the mimetic gold nanoparticles of this size exhibited the best tumor penetration and retention ability. After entering the tumor microenvironment, the hyaluronic acid at the periphery of gold nanoparticles was degraded by hyaluronidase and then internalized and taken up by tumor cells, and the loaded PheoA induced the mass production of intracellular reactive oxygen clusters under NIR irradiation, resulting in the apoptosis and necrosis of tumor cells and the subsequent activation of body’s anti-tumor immune response due to the release of tumor-associated antigen (TAA); meanwhile, the loaded PXTK was hydrolyzed into cinnamaldehyde and PTK under the existence of ROS and played the role of killing tumor cells. Ingeniously, to replenish the ROS consumed by PTXK hydrolysis in time, the hydrolysis by-product cinnamaldehyde could stimulate the production of ROS in mitochondria and promoted the hydrolysis process of PXTK by forming a positive feedback to better exert the anti-tumor effect of PTX. In addition, the team combined the application of anti-PD-L1 peptide (PPA) to alleviate the immunosuppressive microenvironment of tumors, and found that the combination could further enhance the activation of CD4^+^, CD8^+^ T cells, and NK cells by PDT and PTX-induced ICD, and promote the secretion of cytokines such as TNF-α and IL-12 to significantly inhibit tumor growth and metastasis.

## III. Outlook

Inorganic nanomaterials have been widely used in tumor immunotherapy and have achieved good therapeutic effects due to a series of advantages such as high biocompatibility, high targeting ability, immune adjuvant properties, and controllable drug loading and release. It brings the possibility to realize the improvement of symptoms and prognosis of tumor patients.

Although good progress has been made in the study of inorganic nanomaterials for tumor immunotherapy, there are still some shortcomings that need to be focused on. Firstly, to improve the tumor targeting of inorganic nanomaterials and to meet the need of combining multiple therapeutic modalities to achieve the best tumor suppression effect, researchers often need to modify inorganic nanomaterials extensively, which is a tedious process with complicated influencing factors and poor reproducibility, making it difficult to be promoted on a large scale; secondly, the structural basis of inorganic nanomaterials to achieve passive tumor targeting is the enlarged gap between vascular endothelial cells, which makes them leak out from blood vessels to tumor tissue. However, the size of this gap varies greatly among different tumor patients (Ojha et al., 2017), so it is difficult to ensure that the synthesized inorganic nanomaterials can successfully leak out to tumor tissues through the endothelial cell gap among different patients, thus affecting the therapeutic effect. In addition, changes in the local microenvironment of tumors, such as dense extracellular matrix and intra-tumoral high pressure, further limit the infiltration of inorganic nanomaterials into the deeper part of the tumor, making it difficult to achieve the eradication of tumors at the primary site; in recent years, some studies have shown that when inorganic nanomaterials enter the bloodstream, a protein corona will be formed on its surface (Portilla et al., 2021). On the one hand, this structure can affect the dispersion of inorganic nanomaterials in the biological environment, so that they become easily aggregated and do not penetrate well from blood vessels to tumor sites, and on the other hand, this structure can reverse the charge state of positively charged nanoparticles, weakening their ability to be internalized and taken up by tumor cells and immune cells, significantly reducing the effect of tumor immunotherapy (Portilla et al., 2022).

At the same time, based on the the emergence of inorganic nanomaterials, we also call for a demand to conduct deeper research on them from the following four aspects to realize broader application of inorganic nanomaterials in cancer immunotherapy. Firstly, as an efficient carrier of different “cargoes” like tumor antigens, proper inorganic nanomaterials should possess high loading capacity to carry as many “cargoes” as possible. This can be realized through structural optimization of inorganic nanomaterials, and it includes designing nanomaterials of various morphologies to change the contact area between nanomaterials and “cargoes” or cells, resulting in increased amount of carried “cargoes” and enhanced stimulation intensity toward immune system. Secondly, because the detachment of “cargoes” on the nanomaterials or the degradation of them may occur during circulation process in the blood vessel, inorganic nanomaterials with high binding capacity to “cargoes” or hollow structure to involve degradable “cargoes” into cavity should be developed. Thirdly, as an inorganic “non-self” component, inorganic nanomaterials need a comprehensive safety assessment before *in vivo* injection, including injection method, injection dose, duration of action, excretion pathway, and organ toxicity to important organs in the body, etc. Lastly, since the targeting of tumor sites plays a very important role in tumor immunotherapy, which can reduce its systemic toxicity and ensure its biosafety, inorganic nanomaterials with superior targeting ability will be developed in the future by selecting proper synthetic components or surface modifiers based on metabolic characteristics of the tumor microenvironment (e.g. low oxygen, acidic environment, large amount of adenosine secretion, etc.) to improve the efficacy of tumor immunotherapy.

In addition, with the advent of the third generation of nanomaterials—nanorobots, artificial manipulation at nano-level should be considered. Based on asymmetric concentration field, nanorobots can spontaneously target the higher side. Meanwhile, targeting ability of nanorobots can be further enhanced by artificially adjusting the electric potential and the direction of light to change photoelectric field (Dai et al., 2016). This characteristic can also be applied in immunotherapy combined with PDT and its capability to improve tumor targeting precision and therapeutic effects need to be further studied and confirmed. Besides, further research should be carried on to explore more regulating modes to promote the precise and rapid movement toward tumor site.
